# Decision‐analytic models based on real‐world data to assess treatment sequencing in inflammatory bowel disease: A complementary approach to clinical trials

**DOI:** 10.1002/ueg2.12570

**Published:** 2024-04-03

**Authors:** Nicolas Benech, Julien Kirchgesner

**Affiliations:** ^1^ Service d'Hépato‐gastroentérologie, Hôpital de la Croix‐Rousse Hospices Civils de Lyon Lyon France; ^2^ Université Claude Bernard Lyon 1 CRCL Lyon France; ^3^ Department of Gastroenterology Sorbonne Université INSERM, Institut Pierre Louis d’Epidémiologie et de Santé Publique AP‐HP, Hôpital Saint‐Antoine Paris France

A growing number of advanced therapies are available for inflammatory bowel disease (IBD), raising the question of their optimal therapeutic sequence. Few direct head‐to‐head trials exist, and their cost and complexity make unlikely to carry out prospective comparative evaluations of all the various possible therapeutic strategies. Using real‐world data to simulate patient trajectories is a promising strategy to fill knowledge gap.

In ulcerative colitis (UC), vedolizumab, a humanized monocolonal anti‐*α*_4_ *β*_7_, has shown a better efficacy compared to adalimumab in the phase IIIB VARSITY trial.^1^ However, few data are available to determine vedolizumab's position in the therapeutic sequence for Crohn's disease (CD).

In this issue, Louis and collaborators developed a semi‐Markov model to assess how the positioning of vedolizumab in the therapeutic sequence of CD patients can impact patients outcomes.^2^ Markov models are stochastic models that can simulate the evolution of a patient in different simplified states of health (e.g. “health”, “sick”, “dead”), by determining a given transition probability to switch between these different states for a given time period.^3^ Mainly used in health economic studies it can also be developed to address clinical questions.^4^ A common outcome used for Markov‐model‐based studies is quality‐adjusted life‐years (QALYs), that combines health‐related quality of life and life duration and can be considered as an approximation of a criterion combining the effectiveness and safety of a treatment.

In their model, Louis et al, have evaluated different treatment sequences including vedolizumab, corticosteroids, infliximab, adalimumab, and ustekinumab using real‐world data of patients with moderate‐severe CD. In an original setting, the authors also modeled in addition to QALYs the proportion of patients undergoing surgery at 10 years and patient‐reported outcomes (PROs‐based on stool frequency, abdominal pain and wellbeing score).

According to this model, first‐line vedolizumab was more effective based on PROs and outcomes at 10 years than first‐line infliximab (QALYs 5.11 vs. 4.84; patients undergoing surgery: 28.9% vs. 31.9%) or than a first‐line of either infliximab or adalimumab (aggregated data, QALYs 5.09 vs. 4.97; patients undergoing surgery 28.35% vs. 29.85%). Of note, adalimumab was associated with better results when used as a first‐line treatment for both QALYs (5.09 vs. 5.07) and PROs. When comparing the different patient trajectories, vedolizumab as first‐line treatment occupied the optimal position for all types of outcomes assessed.

Markov models can model clinical problems and provide interesting insights when applied properly.^5^, ^6^ In IBD, it has been already used to assess the most cost‐effective sequencing of biologics or the optimal position of vedolizumab in UC.^7^, ^8^ In CD, it has been also used to estimate the probability of treatment maintenance according to the therapeutic sequence when using adalimumab or infliximab.^9^

Several assumptions and simplifications are needed when using Markov models. As discussed in depth by the authors, the external validity of the results may be considered with caution. In particular, the model assumes that all patients are comparable at baseline despite the lack of clinical information to support it. Furthermore, combination therapy and the occurrence of serious infections have not been modeled.

Finally, the reliability of a model highly depends on the incorporated data. Here, a substantial part of the data is based on studies prior to 2010, and transition probabilities, which are essential for the Markov model, were sometimes estimated from a set of just a few patients. However, probabilistic sensitivity analyses were carried out to assess the impact of the uncertain parameters used in the model, and confirmed that the optimal position for vedolizumab was first‐line in 89% of probabilistic simulations when considering QALYs (1780 over 2000 iterations).

Regardless of these limitations, this work provides interesting complementary information to the available data from clinical trials on the vedolizumab. In accordance with these results, post‐hoc analysis of GEMINI trials has also shown that the use of vedolizumab in early disease course was associated with a decreased risk of surgery in CD patients with low/intermediate probability of treatment response.^10^

Thus, further evidence is needed to demonstrate the benefit of first‐line vedolizumab use in CD, but modeling patient trajectories using real‐world data, as performed by Louis and colleagues, is a relevant method for testing and identifying the best therapeutic sequence in IBD in addition to clinical trials.

## CONFLICT OF INTEREST STATEMENT

Nicolas Benech received lecture fees from Tillots Pharma and Mayoly Spindler, travel grants from Pfizer, participated on an advisory board for Ferring Pharmaceuticals with payment to his institution, is a PI of a clinical trial supported by Exeliom Biosciences. Julien Kirchgesner received lecture fees from Galapagos, Janssen and Lilly, and consulting fees from Roche, Pfizer, Janssen, Abbvie, Takeda, Lilly, Celltrion, Tillots, and Galapagos.

## FUNDING INFORMATION

The authors received no financial support for the research, authorship, and/or publication of this article.
